# Supplementary material for: The Impact of Mode of Birth on Childbirth-Related Post Traumatic Stress Symptoms beyond 6 Months Postpartum: An Integrative Review
Source: Int J Environ Res Public Health. 2022 Jul 20;19(14):8830. doi: 10.3390/ijerph19148830 (PMC9316477; doi:10.3390/ijerph19148830)
Supplement: Supplementary file 1 [file ijerph-19-08830-s001.zip › SupTable 1 CASP tool quality assessment quantitative studies.pdf]

Supplementary Table S1: Tool used for the quality assessment of the cohort and cross-sectional studies, based on Critical Appraisal Skills Programme (CASP) checklist for cohort studies and cross-sectional studies by Barnett et al., 2012

12-question checklist	Criteria	Reason for limitation for criteria
1. Did the study address a clearly focused issue?	Initial quality screening questions	
2. Did the authors use an appropriate method to answer their question?		
3. Was the cohort recruited in an acceptable way?	**Criterion 1: External validity**<br>assessment of selection bias	Random sample (transparent, reflects target population, and nonresponse <30% and loss of follow-up <50% = Yes (1 point)<br>Nonrandom sample (not transparent, self-selecting, via Internet or ads) and/or nonresponse >30% and/or follow-up >50% = No (0 points)
4. (a) Was the follow-up of the subjects complete enough?<br>(b) Was the follow-up of the subjects long enough?		
5. Was the exposure (mode of birth) accurately measured to<br>minimize bias?	**Criterion 2: Internal validity**<br>assessment of measurement bias<br>(exposure: mode of birth)	Vaginal birth, operative vaginal birth, emergency caesarean section, elective caesarean section = Yes (1 point)<br>Self-reported after more than 6 weeks = No (0 points)
6. Was the outcome (CB-PTS/D) accurately measured to minimize bias?	**Criterion 3: Internal validity**<br>assessment of measurement bias<br>(outcome: CB-PTS/D)	Validated questionnaire to assess symptoms, interview, diagnosed by a doctor = Yes (1 point)<br>Not-validated questionnaire, using the the word diagnosis 'PTSD' instead of symptoms of PTSD = No (0 points)
7. (a) Have the authors identified all important confounding factors?<br>(b) Have they taken account of the confounding factors in the design and/or analysis?	**Criterion 4: Internal validity**<br>assessment of confounding	Studies not calculating associations:<br>Characteristics or measures for previous trauma (e.g. sexual abuse) and mental health (e.g. depression, anxiety) Yes = (1 point)<br>No characteristics or measures for previous trauma (e.g. sexual abuse) or mental health (e.g. depression, anxiety) No = (0 points)<br><br>Studies calculating associations:<br>Adjusted for confounders: previous trauma (e.g. sexual abuse) and mental health (e.g. depression, anxiety) = Yes (1 points)<br>Adjusted for only one or none of the confounders = No (0 points)
8. What are the results of this study?	Overall quality assessment of the results and transferability of the study findings (as determined by likelihood of selection bias (Criterion 1); measurement biases (Criteria 2 and 3); and confounding (Criterion 4).	
9. How precise are the results?		
10. Do you believe the results?		

	No additional quality criterion was defined	
11. Can the results be applied to the local population?		
12. Do the results of this study fit with other available evidence?		
