# Supplementary material for: The Impact of Mode of Birth on Childbirth-Related Post Traumatic Stress Symptoms beyond 6 Months Postpartum: An Integrative Review
Source: Int J Environ Res Public Health. 2022 Jul 20;19(14):8830. doi: 10.3390/ijerph19148830 (PMC9316477; doi:10.3390/ijerph19148830)
Supplement: Supplementary file 1 [file ijerph-19-08830-s001.zip › SupTable 2 CASP qualitative studies.pdf]

Supplementary Table S2: Quality assessment of the included qualitative studies based on Critical Appraisal Skills Programme (CASP) Methodology

Studies	1. Was there a clear statement of the aims of the research?	2. Is a qualitative methodology appropriate?	3. Was the research design appropriate to address the aims of the research?	4. Are the study's theoretical underpinnings clear, consistent and conceptually coherent?	5. Was the recruitment strategy appropriate to the aims of the research?	6. Was the data collected in a way that addressed the research issue?	7. Has the relationship between researcher and participants been adequately considered?	8. Have ethical issues been taken into consideration?	9. Was the analysis sufficiently rigorous?	10. Is there a clear statement of findings?
Ayers et al. 2006	Yes	Yes	Yes	Yes	Yes	Yes	Can't tell	Yes	Yes	Yes
Beck et al., 2004	Yes	Yes	Yes	Yes	Yes	Yes	Can't tell	Yes	Yes	Yes
